# Comparative analysis of the oral mucosae from rodents and non-rodents: Application to the nonclinical evaluation of sublingual immunotherapy products

**DOI:** 10.1371/journal.pone.0183398

**Published:** 2017-09-08

**Authors:** Catherine Thirion-Delalande, Frédéric Gervais, Cécile Fisch, Jean Cuiné, Véronique Baron-Bodo, Philippe Moingeon, Laurent Mascarell

**Affiliations:** 1 CiToxLAB, Evreux, France; 2 Technical Development Department, Stallergenes Greer, Antony, France; 3 Research Department, Stallergenes Greer, Antony, France; Centre National de la Recherche Scientifique, FRANCE

## Abstract

**Background:**

A comparative characterization of the oral mucosa in various animals is needed to identify the best animal model(s) for nonclinical evaluation of sublingual immunotherapy products. With this aim, we studied the histological characteristics and immune cell infiltrates of oral mucosae from common animal species.

**Methods:**

Three oral regions (*i*.*e*. ventral surface of the tongue, mouth floor and cheek) obtained from eight animal species, including rodents (*i*.*e*. mice, rats, hamsters, guinea pigs) and non-rodents (*i*.*e*. rabbits, dogs, minipigs and monkeys) were characterized by histology and immunohistology in comparison with a human tongue.

**Results:**

Rodents exhibit a thin keratinized epithelium with low epithelial extensions, whereas non-rodents, most particularly minipigs and monkeys, display a non-keratinized epithelium with larger rete ridges, similarly to humans. Glycogen-rich cells in the superficial epithelial layers are observed in samples from both minipigs, monkeys and humans. Comparable immune subpopulations detected in the 3 oral regions from rodent and non-rodent species include MHC-II^+^ antigen presenting cells, mostly CD163^+^ macrophages, located in the *lamina propria* (*LP)* and muscle tissue in the vicinity of resident CD3^+^CD4^+^ T cells. Limited numbers of mast cells are also detected in the *LP* and muscle tissue from all species.

**Conclusion:**

The oral mucosae of minipigs and monkeys are closest to that of humans, and the immune networks are quite similar between all rodents and non-rodents. Taking into account the ethical and logistical difficulties of performing research in the latter species, rodents and especially mice, should preferentially be used for pharmacodynamics/efficacy studies. Our data also support the use of minipigs to perform biodistribution and safety studies of sublingual immunotherapy products.

## Introduction

Sublingual allergen immunotherapy (AIT) is currently established as a safe and efficient therapy for type I respiratory allergies, both in adults and children [1–11]. Immune mechanisms underlying AIT efficacy are well-documented and involve a switch from Th2 to Th1/Treg responses in the blood and in targets organs [12,13]. In light of the growing interest for sublingual AIT, there is a need for animal models to test candidate products. Biodistribution and pharmacodynamics (PD) studies conducted in such nonclinical models have shown that allergens are detected at the mucosal surface of the tongue within a 2-hour period after administration and are not directly absorbed into the bloodstream [14–17]. Rather, allergens cross the oral mucosa to accumulate at the mucosal/submucosal junction prior to being captured by resident professional antigen presenting cells (APCs), which subsequently migrate to draining cervical lymph nodes to elicit allergen-specific Th1/Treg responses [12,13]. These oral APCs, located in the upper layers of oral tissues, include CD207^+^ Langerhans cells (LCs) present in the epithelium, as well as CD11b^+^/CD68^+^/CD206^+^ macrophages located along the *lamina propria* [16–27]. Of note, these two allergen-capturing APC subsets exhibit a tolerogenic phenotype, in that they express anti-inflammatory factors *i*.*e*. RALDH, IDO, IL-10 and TGFβ, likely contributing to inducing allergen-specific peripheral tolerance.

In the aforementioned studies, murine models have been established as valuable tools for investigating the mode of action of AIT, in light of evidence suggesting an overall comparable immune system to that of humans. However, the use of alternative animal models appropriate for PD/efficacy studies can also be considered. Also, mice may not be the best animal model for biodistribution studies due to a different mucosal architecture when compared to humans [28,29]. For those reasons combined, the identification of animal models to mimic optimally the human physiology is of interest for the nonclinical evaluation of new sublingual AIT products.

To this aim, we conducted a comprehensive study of oral tissues (encompassing the ventral surface of the tongue, mouth floor and cheek) obtained from eight animal species (including mice, rats, hamsters, guinea pigs, rabbits, dogs, minipigs and monkeys) in comparison with human tissues by using histology and immunohistology analyses. Herein, beyond the relative merit of the various animal models, we provide specific evidence for the interest of murine and minipig models to document the efficacy as well as the biodistribution and safety, respectively, of allergens intended for sublingual AIT.

## Materials and methods

### Animal and human samples

Characteristics of the animals used in this study are described in Table 1 and international levels of ethical standards were applied for animal handling. This study (internal number 40573 EP: “histology of mouth mucosa (sublingual region): inter-species evaluation” was conducted for research purposes. The mouth floor, sublingual and cheek mucosae from several animal species had been sampled and examined microscopically. Since the sampling procedure was invasive, the animals had to be euthanized prior to the sampling. Simple biopsy punch could not be performed. This allowed the examination of the epithelium but also of the deep lamina propria, including the vasculature of these areas. A human tongue was purchased from Tissue Solutions Ltd (Glasgow, United Kingdom).

**Table 1:** Characteristics of animals used in the experiments.

Species	Strain	sex	Age	Supplier	Sacrifice method
Mouse	129/SvEv	M (n = 3)	12 w	Taconic, Denmark	The animals (mice, rats, hamsters, Guinea pigs and rabbits) were deeply anesthetized by an intraperitoneal injection of sodium pentobarbital and sacrificed by exsanguination.
Rat	Sprague-Dawley CD^®^ IGS	M (n = 3)	10 w	Charles River Laboratories, Italia	
Hamster	Gold (*Mesocricetus auratus*)	M (n = 3)	4–5 w	Janvier, France	
Guinea pig	Hartley	F (n = 3)	6 w	Charles River, France	
Rabbit	New Zealand White	M (n = 3)	12 w	CEGAV, France	
Dog	Beagle	M (n = 3)	16 m	CEDS, France	Dogs were sedated by an intramuscular injection of a mixture of tiletamine and zolazepam, anesthetized by an intravenous injection of pentobarbital sodium and euthanized by exsanguination.
Monkey	Cynomolgus (*Macaca fascicularis*)	M (n = 3)	42–47 m	Le Tamarinier, Mauritius	Monkeys were tranquilized by an intramuscular injection of ketamine hydrochloride, anesthetized by an intravenous injection of pentobarbital sodium and sacrificed by exsanguination.
		F (n = 1)	5.5 y	Nafovanny, Vietnam	
Minipig	Göttingen	M (n = 3)	10–14 m	Ellegaard, Denmark	Minipigs were deeply tranquillized by an intramuscular injection of a mixture of tiletamine and zolazepam, anesthetized by an intravenous injection of pentobarbital sodium and sacrificed by exsanguination

Live animals were obtained by the listed suppliers. This research work was performed on tissues that were sampled at necropsy on animals that were already euthanized for other purposes. There were not euthanized expressly for the intended work. This procedure contributed to the reduction promoted by the 3Rs. CiToXLAB France is accredited by AAALAC and all the animals are properly housed, cared and used in compliance with the current rules and regulations including the Directive 2010/63/EU and the corresponding French lawsThree animals were used for each species. M: male; F: female; w: weeks; m: months; y: years. CEGAV: Centre d’élevage Gontran Achard de la Vente. CEDS: Centre d’élevage du domaine des souches

### Tissue preparation and histology analyses

The head of rodents (*i*.*e*. mice, rats, hamsters and guinea pigs), half of the head (longitudinally-cut) of non-rodents (*i*.*e*. rabbits, dogs, minipigs and monkeys) and the human tongue were fixed in phosphate buffered formalin-zinc (Microm microtech, Brignais, France), decalcified and embedded in paraffin blocks. The other samples were snap frozen. Tissue sections (5 μm thick) were transversally cut, mounted on glass slides (Tissue-Tek® AutoWrite® Starfrost® Adhesion microscope slides, Sakura Finetek Europe B.V, Alphen-sur-le-Rhin, Netherlands) and stained with either hematoxylin-eosin (HE) and Masson trichrome (Microm microtech) to analyze the structure of the mucosa, or with toluidine blue (Microm microtech) to quantify mast cells. Additional tissue sections obtained from mucosae of mice, minipigs, monkeys and humans, for which a clear cytoplasm in epithelial cells is found, were stained with Period Acid Schiff (PAS) (Microm microtech) to evaluate the presence of glycogen. To prove the glycogen nature, PAS staining used in combination with diastase (PAS diastase) allowed glycogen to be degraded to ensure the specificity of the assay.

For each tissue section, the structure of the mucosa obtained from either the ventral surface of the tongue, the mouth floor or the cheek was analyzed with respect to (1) the presence or absence of keratin, (2) the numbers of epithelial layers, (3) the shape of epithelial extensions *i*.*e*. rete ridges graded as absent, minimal, slight, moderate or marked, (4) the density of connective tissues considered as loose, intermediate or dense according to the brightness of Masson trichrome staining, a blue dye used for the detection of collagen fibers, (5) the number/size of vessels in the *lamina propria* (*LP*) graded as slight, moderate or marked.

### Tissue preparations for immunohistology analyses

Mucosal tissues previously snap-frozen and stored in a freezer at -80°C were included in Tissue-Tek® O.C.T.™ Compound (Sakura Finetek Europe B.V). Tissue sections (5 μm thick) were serially cut, mounted on glass slides (Tissue-Tek® AutoWrite® Starfrost® Adhesion microscope slides, Sakura Finetek Europe B.V), air-dried at room temperature (RT) and fixed in a solution of 5% Zinc Formalin Fixative (Microm microtech), prior to rehydration. In selected analyses (*i*.*e*. rat tissue sections labelled with anti-CD4 or CD172a mAbs and minipig tissue sections labelled with anti-CD172a mAbs), slides were fixed in acetone (Carlo Erba, Val de Reuil, France) supplemented with 10–30% methanol (Carlo Erba). Slides were uploaded into the Ventana Discovery Ultra system (Ventana Medical Systems, Tucson, AZ), and an automated immunohistochemical staining was performed. For tissue sections from dogs, minipigs and monkeys labelled with anti-CD3 mAbs or monkey tissue sections labelled with anti-CD172a mAbs, an antigen retrieval step was carried out by heating at 95°C and incubating samples for 4 minutes in a Tris-based buffer with a slightly basic pH (Cell conditioning 1 from Ventana Medical systems). After blocking both endogenous biotin (*i*.*e*. with avidin/biotin reagents, Ventana Medical Systems) and peroxidase activity with a 3% H_2_O_2_ methanol solution (Carlo Erba), tissue sections were incubated for one hour at 37°C with primary antibodies (including matching isotype control antibodies) diluted in Dako diluent at concentrations between 0.2 to 10 µg/mL. After washing, tissue sections were incubated for 30–45 minutes with appropriate biotin-conjugated goat anti-mouse (Abcam), anti-rat (Abcam) or anti-rabbit (Jackson ImmunoResearch, Suffolk, UK) antibodies prepared at 1/200 dilution. Tissue sections were washed, and specific staining was visualized after incubation with a diaminobenzidine substrate solution (DAB Map kit, Ventana Medical Systems) according to the manufacturer’s recommendations. A purple chromogen detection system (UltraMap Anti-Mouse HRP + Purple Kit Discovery, Ventana) was preferred to detect CD163 positive cells from dog and monkey tissue sections in order to distinguish them from melanin pigments in the mucosa. For cell quantification, samples were scored blindly. Countings were performed on at least 5 fields per area under a light microscope (Olympus BX51, Olympus Optical Co., Tokyo, Japan) using a 40x objective lens and 10x eyepiece lenses. The mean number of positive cells/field for a given immune marker was calculated at the level of the epithelium, *Lamina propria* and muscle for each region in tissue samples obtained from all animal species.

### Antibodies

The following polyclonal or monoclonal antibodies (mAbs) were used for immunohistology: anti-CD3 (for rats: clone 1F4, Bio-Rad, Oxford, UK; for dogs: Ab828, Abcam, Cambridge, UK; for minipigs: clone 8E6, WSU Monoclonal antibody center, Pullman, WA; for monkeys: clone CD3-12, Abcam), anti-CD4 (for rats: clone OX-35, Bio-Rad; for dogs: clone DH-29A, WSU Monoclonal antibody center; for minipigs: clone 74-12-4, WSU Monoclonal antibody center; for monkeys: clone BC/1F6, Abcam), anti-CD163 (for all species: clone AM-3K, Antibodies online, Paris, France), anti-CD172a (for rats: clone ED9, Bio-Rad; for dogs: clone DG-DH59B, WSU Monoclonal antibody center; for minipigs: clone BL1H7, Bio-Rad; for monkeys: Ab139698, Abcam), anti-MHC-II (for rats: clone OX-6, Bio-Rad; for dogs: clone DG-H42A, WSU Monoclonal antibody center; for minipigs: clone TH21A, WSU Monoclonal antibody center; for monkeys: clone L243, Abcam). Corresponding isotype-matched mAbs were used as controls in all experiments.

## Results

### Compared histology of oral mucosae in animal species and humans

We first analyzed by histology mucosae retrieved from the oral regions relevant in the context of sublingual administration including *i)* the ventral surface of the tongue, *ii)* the mouth floor and *iii)* the cheek as shown in S1 Fig. Specifically, various structural components, likely influencing the biodistribution of allergens, were analyzed in these mucosae. The latter include the presence of keratinized cells, the number of epithelial cell layers, the presence of epithelial extensions such as rete ridges, the density of connective tissue in the *lamina propria* (*LP)* and the mumbers and size of blood vessels. Results of these analyses conducted in samples from each animal species are summarized in Table 2.

**Table 2:** Overview of histological features of oral mucosae.

Species	Area	Keratin	Numbers of epithelial layers	Rete ridges	Connective tissue density	Vascularization
Mouse	Ventral surface	+	6–8	-	+++	+++
	Mouth floor	+	6–8	+/-	++	++
	Cheek	+	8–12	++	++	++
Rat	Ventral surface	+	8–12	++	+++	+++
	Mouth floor	+	6–8	++	++	++
	Cheek	+	12–18	+	++	++
Hamster	Ventral surface	+	3–10	+/-	+++	+
	Mouth floor	+	6–12	-	+++	++
	Cheek	+	3–6	+/-	+++	++
Guinea pig	Ventral surface	+	12–16	++	++	++
	Mouth floor	+	12–20	+	+	+++
	Cheek	+	20–30	++	++	++
Rabbit	Ventral surface	-	20–30	+/-	+++	++
	Mouth floor	-	15–25	+	++	++
	Cheek	-	30–40	+/-	+++	+
Dog	Ventral surface	-	8–12	+/-	+++	+++
	Mouth floor	-	12–18	+	+	+++
	Cheek	-	15–25	++	++	+++
Minipig	Ventral surface	-	15–20	+++	+++	++
	Mouth floor	-	10–20	+++	+	+++
	Cheek	-	30–40	+++	++	+++
Monkey	Ventral surface	-	20–30	++	+++	++
	Mouth floor	-	20–30	++	++	+++
	Cheek	-	30–40	++	+++	++
	Ventral surface	-	20–30	++	+++	+++
Human	Mouth floor	NA	NA	NA	NA	NA
	Cheek	NA	NA	NA	NA	NA

Histology of oral tissues (*i*.*e*. the ventral surface of the tongue, the mouth floor and the cheek) from eight animal species (*i*.*e*. mice, rats, hamsters, guinea pigs, rabbits, dogs, minipigs and monkeys with n = 3 for each species) was performed following staining with either Hematoxylin-Eosin (HE) or Masson trichrome. As well, one human tongue was analyzed in a similar way to compare with these animal tissues. The structure of the mucosa was analyzed according to: i) the presence (+) or absence (-) of keratin, ii) the numbers of epithelial layers, iii) the shape of epithelial extensions *i*.*e*. rete ridges graded as absent (-), minimal (+/-); slight (+), moderate (++) or marked (+++), iv) the density of connective tissues considered as loose (+), intermediate (++) or dense (+++) and v) the number/size of vessels in the *lamina propria* (LP) graded as slight (+), moderate (++) or marked (+++). NA means not available.

**Fig 1:**
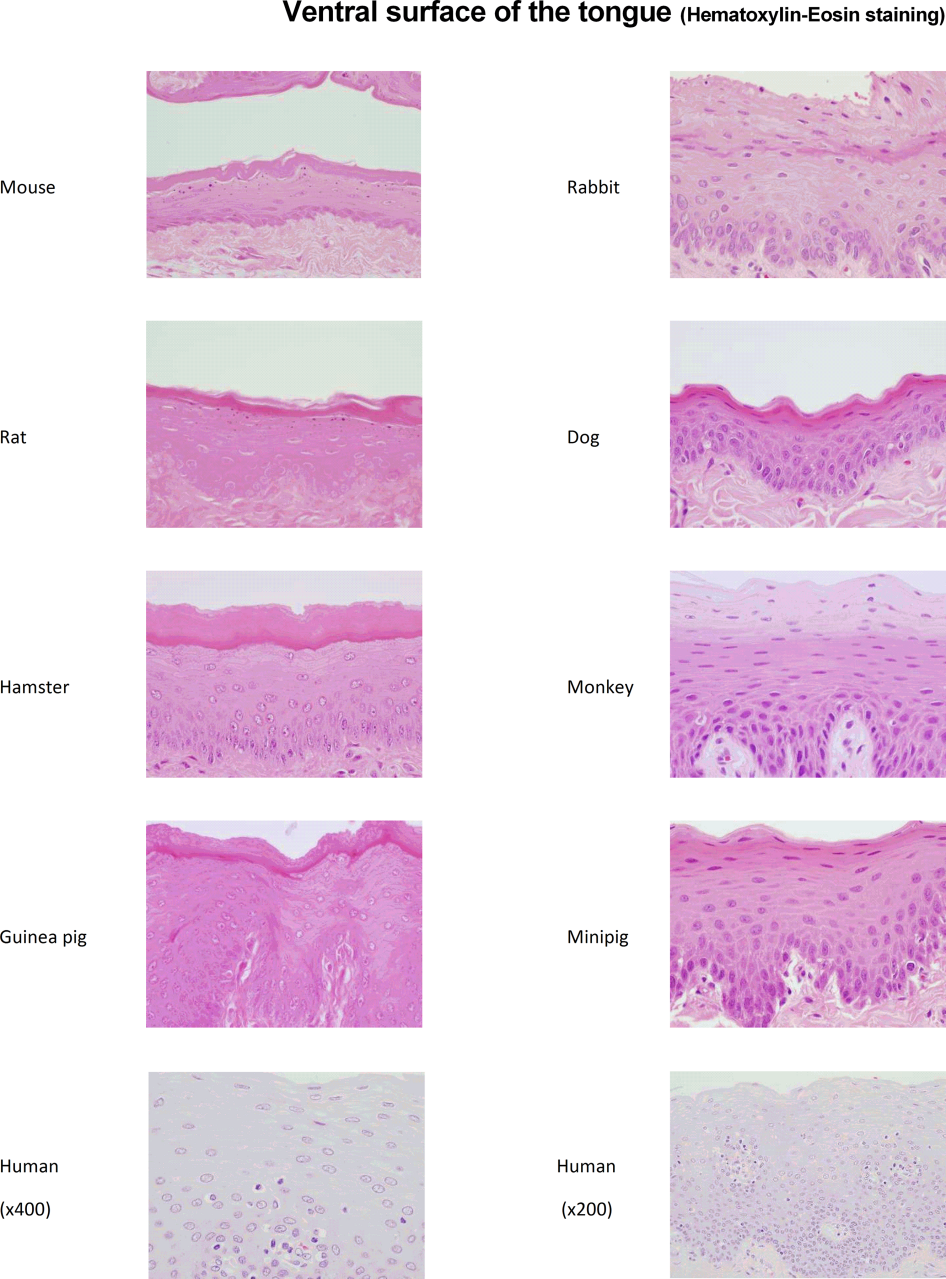
Histology of the ventral surface of the tongue from rodents, non-rodent species and humans. Representative photomicrographs of mucosal (*i*.*e*. ventral surface of the tongue) tissue sections (magnification x400 for animal species and x200/x400 for human) embedded in paraffin and stained with Hematoxylin-Eosin (HE) are shown.

**Fig 2:**
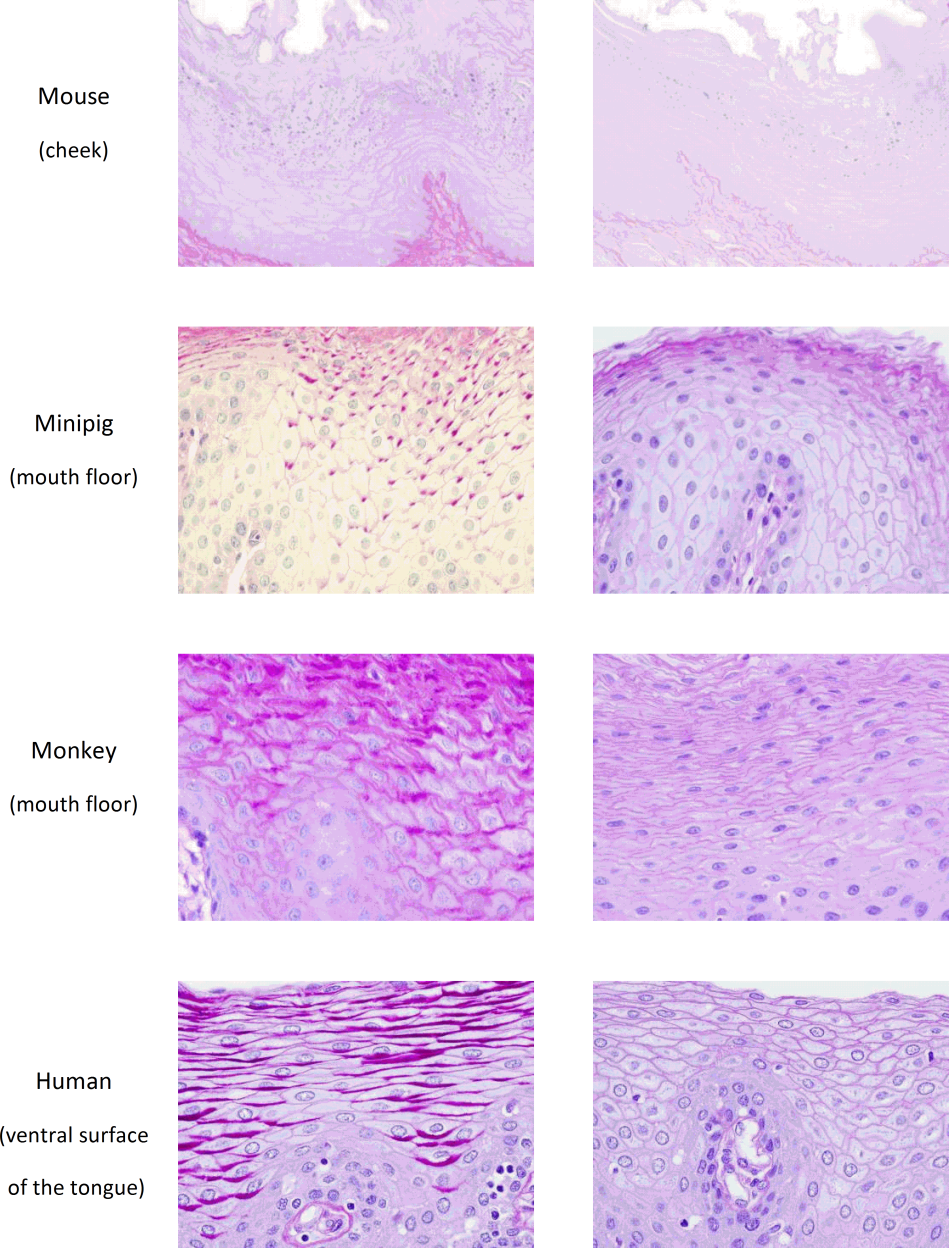
Detection of glycogen in the oral mucosae of mice, minipigs, monkeys and humans. Representative photomicrographs of mucosal (*i*.*e*. ventral surface of the tongue, mouth floor or cheek as indicated in the figure) tissue sections (magnification x400) embedded in paraffin and stained with PAS and PAS diastase are shown.

The keratinization of the epithelium was mainly observed in rodent species (*i*.*e*. mice, rats, hamsters and guinea pigs) in all the 3 examined mucosal regions (Fig 1 and S2 and S3 Figs), even if a clear superficial cell zone devoid of keratin was observed in the cheek of mice (S3 Fig). Conversely, non-rodent species (*i*.*e*. rabbits, dogs, minipigs and monkeys) display a non-keratinized mucosal lining similar to the one observed in humans, with the exception of a tiny keratinized area localized in the epithelium from the cheek of rabbits (S3 Fig). The presence of glycogen was also evaluated in the non-keratinized epithelial cells. In mice, the focal cheek region devoid of keratin (S3 Fig) was not stained following the PAS reaction, indicating that these cells do not contain any glycogen (Fig 2). Conversely, in minipigs, monkeys and humans, cells located in the upper half of the epithelium were positively PAS-stained (Fig 2), demonstrating the presence of a glycogen-rich content, thus providing evidence for similarities of oral mucosae between minipigs, monkeys and humans.

The thickness of the epithelium, *i*.*e*. the numbers of epithelial layers, as well as the depth of rete ridges, are generally related to the size of the species, as observed in the 3 mucosal regions (Fig 1, S2 and S3 Figs and Table 2). In rodents, hamsters have the thinnest epithelium with 3 to 12 cell layers, whereas mice and rats exhibit 6 to 18 cell layers in their epithelium. Noteworthy, the guinea pig has the thickest epithelium with 12 to 30 cell layers. In non-rodents, the numbers of layers are higher. Whereas dogs have the thinnest epithelium with 8 to 25 cell layers, the epithelia of rabbits, minipigs and monkeys comprise 15 to 40 cell layers, similarly to that of humans. The rete ridges are thin in rodents and are even flat in the ventral surface of the tongue of mice or in the mouth floor of hamsters. In non-rodent species, rabbits and dogs exhibit limited epithelial extensions while minipigs, monkeys, and humans show longer rete ridges in the epithelium (Table 2).

**Fig 3:**
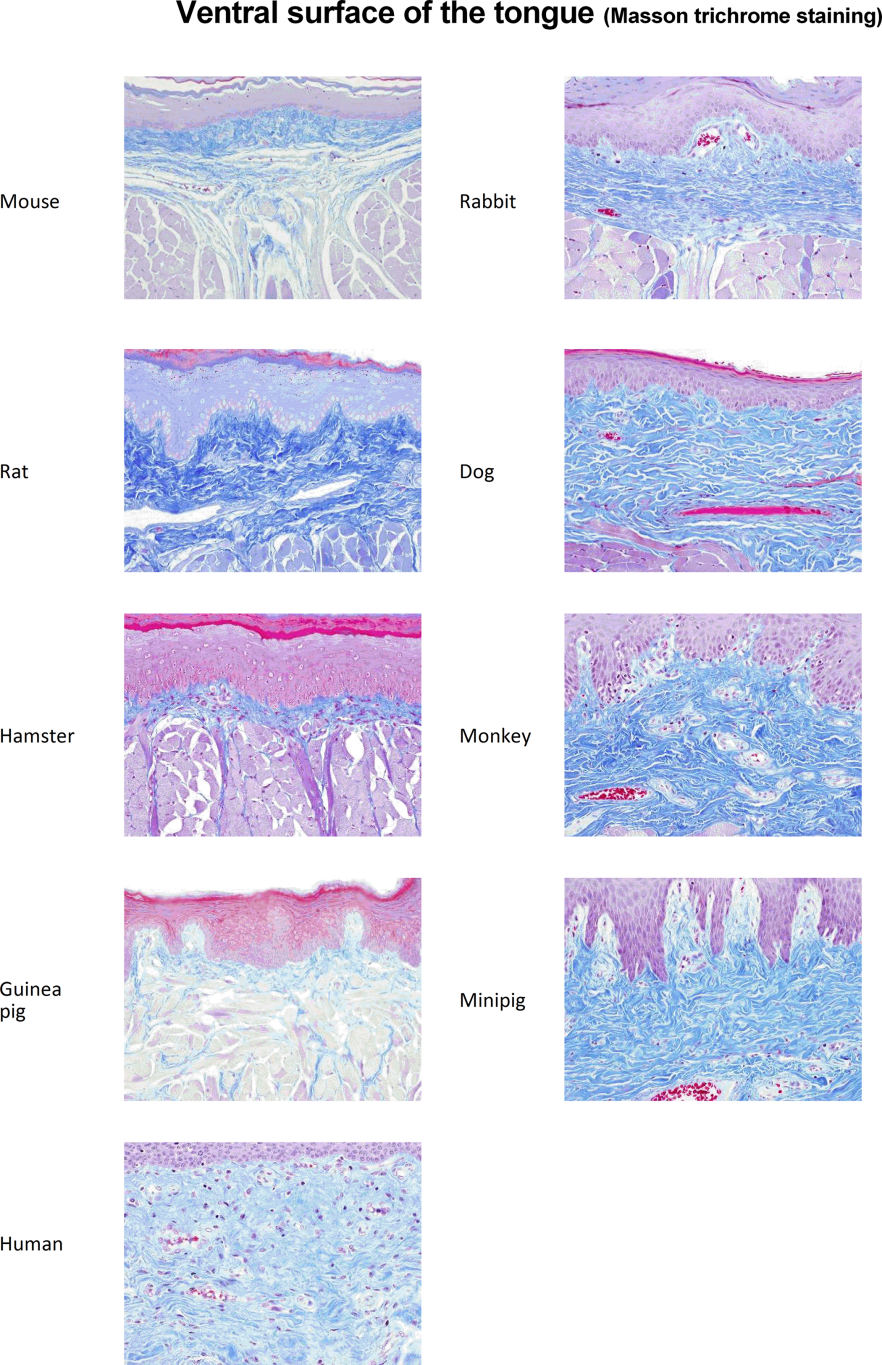
Collagen fibers density in the mucosa of the ventral surface of tongues from rodents, non-rodent species and humans. Representative photomicrographs of mucosal (*i*.*e*. ventral surface of the tongue) tissue sections (magnification x200) embedded in paraffin and stained with Masson trichrome are shown.

The connective tissue density of LP is more pronounced in the ventral surface of the tongue when compared with the mouth floor or the cheek, but no major differences are seen in this regard between animal species and humans (Fig 3, S4 and S5 Figs and Table 2). As well, the vascular network is overall more developed in the tongues of mice, rats, dogs and humans, but remains close to that observed in other species *i*.*e*. guinea pigs, rabbits, minipigs and monkeys (Table 2).

Consequently, parameters such as connective tissue density and numbers/size of blood vessels cannot be used as a discriminating criterion among species. However, based upon three characteristics of the oral epithelium, *i*.*e*. keratinization, thickness and rete ridges, oral mucosae from both minipigs and monkeys are the ones exhibiting the closest similarities with that of humans. As such, these data suggest the interest of the aforementioned non-rodent species for studying the biodistribution as well as safety of allergen products intended for sublingual administration.

### Mapping of oral immune cells in rats, dogs, minipigs and monkeys

**Fig 4:**
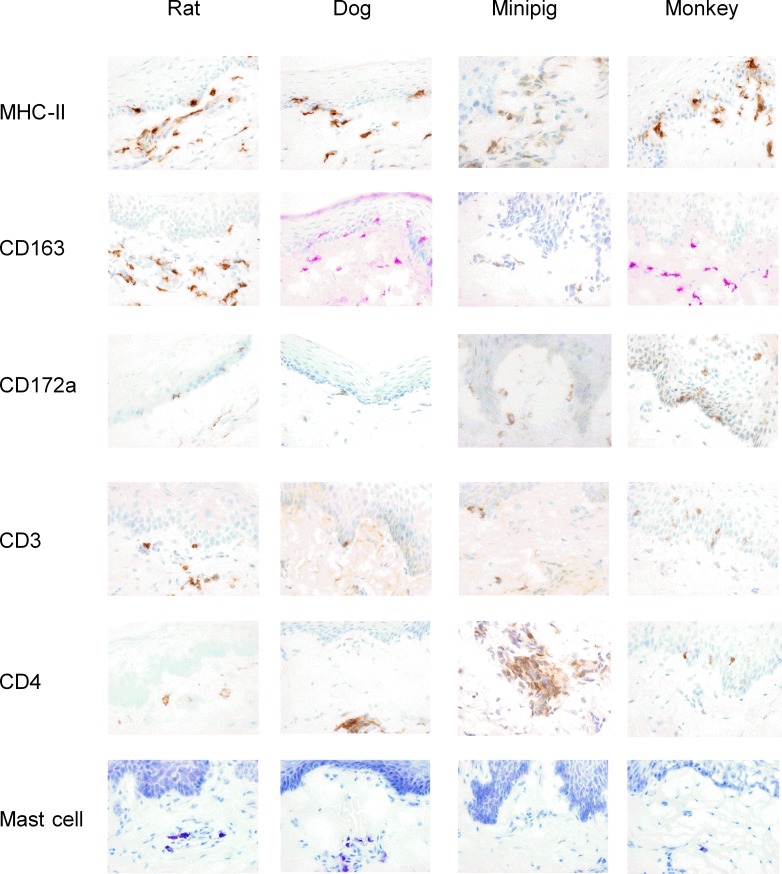
Mapping of immune cells in the ventral surface of tongues from rats, dogs, minipigs and monkeys. Mucosal tissues from animal species were processed for immunohistology analysis, as described in Methods. Slides were stained with the following specific antibodies: anti-MHC-II, anti-CD163, anti-CD172a, anti-CD3, and anti-CD4 to detect and quantify positive cells in tissue sections or with toluidine blue to quantify mast cells (magnification x200). Representative photomicrographs of mucosal tissue sections are shown.

**Fig 5:**
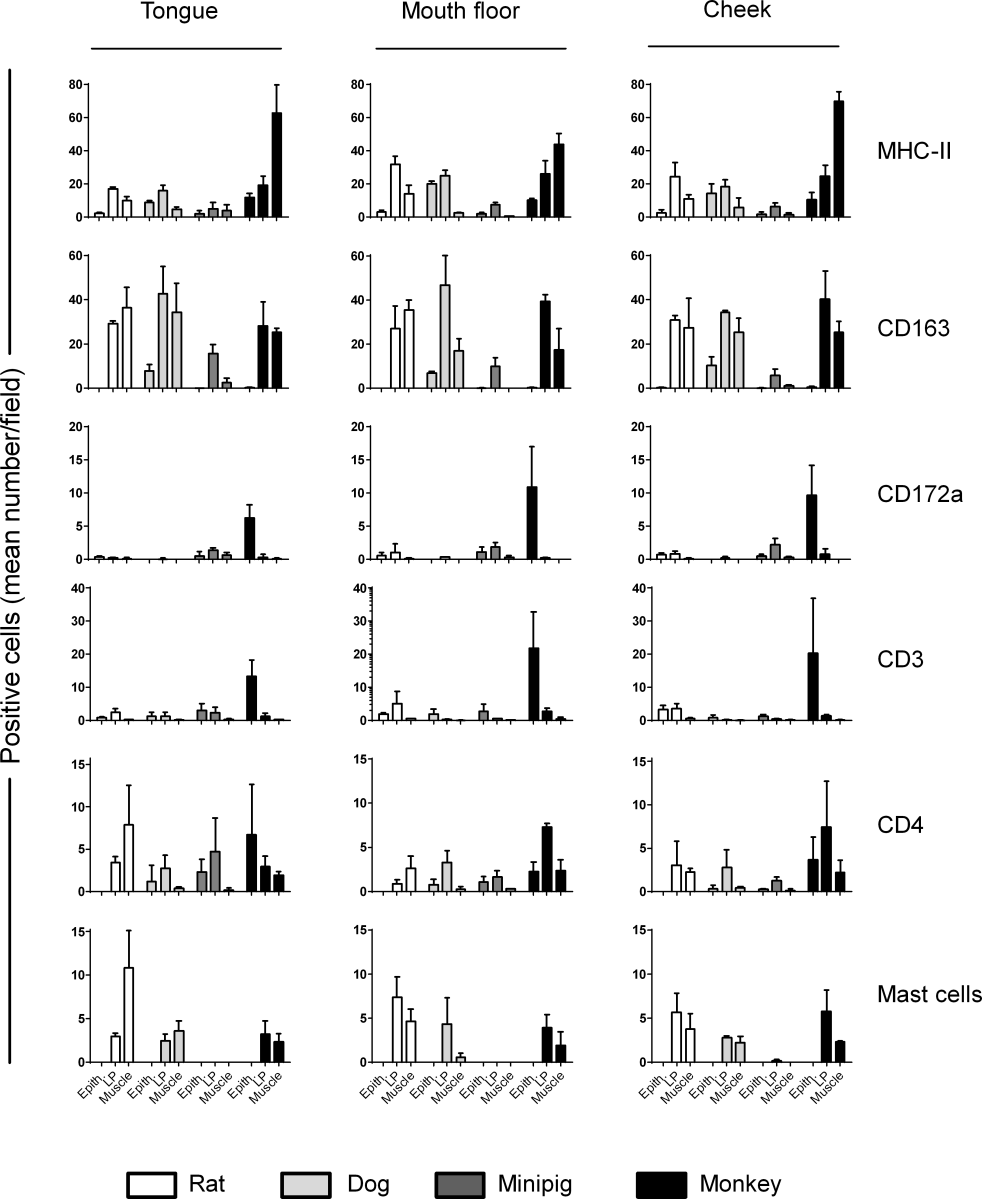
Quantification of immune cells in the ventral surface of the tongue, the mouth floor and the cheek from rats, dogs, minipigs and monkeys. Mucosal tissues from animal species were processed for immunohistology analysis, as described in Methods. Cell counting was performed on slides labeled with Abs specific for APC (anti-MHC-II, anti-CD163, anti-CD172a) and T cell (anti-CD3 and anti-CD4) markers or stained with toluidine blue for mast cells to evaluate the mean number of positive cells per field using a light microscope (magnification x400). All areas (epithelium (Epith.), *Lamina propria* (LP) and muscle) were scored. Histograms represent the mean + SEM with n = 3.

The phenotype and tissue distribution of immune cells were subsequently assessed by immunohistology at 3 different sites (*i*.*e*. Epithelium (Epith.), LP and muscle) from the ventral surface of the tongue, the mouth floor and the cheek of 4 animal species including one rodent (rat) and three non-rodent species (*i*.*e*. dog, minipig and monkey) sharing similar histological features with the human mucosa. A panel of specific antibodies against surface markers associated either with antigen presenting cells (MHC-II), macrophages (CD163), Langerhans/dendritic cell (CD172a) or T cells (CD3, CD4) were used to detect and quantify in tissue sections immune cells involved in AIT mechanisms, as described in Methods. For the detection and quantification of mast cells, tissue sections were stained with toluidine blue. Qualitative and quantitative results are presented in Figs 4 and 5, respectively.

MHC-II+ antigen presenting cells (APCs) are mainly detected in the LP of the 3 mucosal regions in all species studied except for monkey exhibiting higher numbers of MHC-II+ APCs in the muscle. Strikingly, although clearly detectable, MHC-II+ APCs are less abundant in minipig than in other species in all the 3 mucosal regions. Most of MHC-II+ APCs encompass CD163+ macrophages located in the LP and muscle tissue, but also some Langerhans/dendritic cells. In particular, CD172a+ cells, which likely correspond to LCs, were mainly detected in the epithelium of monkeys. Oral resident CD4+ T cells are also predominantly located in the LP of the 3 mucosal regions in all species, in close vicinity with MHC-II+ APCs. Interestingly, a large number of CD3+ (CD4-) cells, likely CD3+CD8+ cells, are detected only in the epithelium of monkeys. Lastly, a low numbers of mast cells are located only in deeper tissues (*i*.*e*. LP and muscle tissue) and are barely detectable in minipigs. As a whole, our analyses confirm that the immune system is similar in oral samples from all species, suggesting that multiple animal models can be used for PD/efficacy studies of sublingual AIT products.

## Discussion

Immune mechanisms taking place in the oral mucosa following sublingual allergen administrations have been addressed in several studies by using both in vitro human and in vivo murine models, leading to converging observations [16–27]. In particular, oral APCs, mainly Langerhans cells (LCs) and macrophages have been shown to be critical to capture allergens and to support the development of allergen-specific Th1/Treg immune responses [16–19,30]. However, due to potential differences between the oral mucosae in rodents and humans, selected animal models better mimicking human conditions may be more appropriate for the nonclinical (*i*.*e*. biodistribution, safety and efficacy) evaluation of sublingual AIT products under development.

Herein, we undertook a detailed histology study as well as a mapping of immune cells in oral tissues (*i*.*e*. ventral surface of the tongue, mouth floor and cheek) obtained from eight animal species (*i*.*e*. mice, rats, hamsters, guinea pigs, rabbits, dogs, minipigs and monkeys), in comparison with the human oral mucosa. Our analyses established that three features of the oral epithelium, *i*.*e*. keratinization, thickness and rete ridges, represent interesting discriminating criteria between animal species and humans, inasmuch as differences at this level may significantly alter the allergen capture in the course of sublingual AIT. Specifically, the pronounced keratinization of the superficial layer of the oral mucosa in rodents may result in a possible physical barrier that minimizes the penetration of allergens in the lower epithelium and LP. Conversely, a thin epithelium may rather favor a passage of allergens toward blood vessels in the LP, impeding an optimal capture by mucosal resident APCs. In this respect, we document herein that non-rodent species, most particularly minipigs and monkeys, exhibit a thick non-keratinized mucosal lining with large epithelial extensions, similarly to what is observed in humans. In addition, the presence of a glycogen-rich content in the cytoplasm of epithelial cell detected only in minipigs and monkeys also supports the inference that such cells are similar to those of humans. In line with these observations, several studies conducted in allergy murine models showed that higher doses (fifty to a hundred times more) of allergen are required for a successful treatment in allergic mice when compared to doses administered to humans in the course of AIT [31–37]. Consequently, such models only approximate the human situation regarding allergen dosage and administration schemes. As a whole, our histological evaluation demonstrates that the oral mucosae from minipigs and monkeys exhibit similar histological features with that of humans. For housing, care and ethical purposes, we propose that minipig-based animal models should be preferred for studying allergen biodistribution following sublingual administration. Minipigs are increasingly used in toxicology studies given their anatomy and physiology comparable to human [38,39]. As allergy models, they have been previously used in some studies [40,41]. Specifically, minipigs sensitized to peanut-allergens exhibit clinical symptoms closely related to human allergic responses, including skin rashes, vomiting, lethargy, convulsions, respiratory distress, diarrhea as well as anaphylactic shocks. These findings along with our observations confirm that minipigs share histologic and physical features with humans, suggesting a clear interest of minipigs as toxicology models for sublingual AIT products.

Another goal of our study was to characterize the immune system of the oral mucosa in animal species for which little information is available as of today. Mapping of immune cells in oral tissues from both rodent (*i*.*e*. rat) and non-rodent species (*i*.*e*. dogs, minipigs and monkeys) confirms previous studies in BALB/c mice and humans concerning the immune networks within the oral mucosa [16,21,27]. Most particularly, APCs, *i*.*e*. dendritic cells and macrophages, are mainly located in the epithelium and LP of the oral mucosa in the proximity of oral resident CD3+ T cells, suggesting that adaptive immune responses to antigens/allergens can be, to some extent, carried out locally. As well, the presence of macrophages expressing CD163, a scavenger receptor contributing to the regulation of immune responses [42–44], may reflect the bias of such macrophages to promote tolerance induction upon allergen capture, thus confirming our previous observations on the tolerogenic function of oral macrophages in mice and humans [17,27]. As well, very low numbers of mast cells present only in deeper tissues of the studied species strenghten our previous observations made in mice and humans that the contact between allergen(s) and pro-inflammatory cells is very limited in the oral mucosa, likely explaining the good tolerability of sublingual AIT [20,21,27,45]. Altogether, our analyses of the immune system in rodents and non-rodents did not reveal major qualitative differences between species and suggest that all species tested could possibly be used for PD/efficacy studies.

Collectively, our findings establish that some differences exist in oral tissues between animal species likely affecting the uptake and fate of allergen(s) following sublingual administration. Our observations clearly support the use of minipigs for studying biodistribution as well as safety of new sublingual AIT products while the use of rodents (e.g. mice), for practical and ethical reasons, remains appropriate for the nonclinical assessment of PD/efficacy.

## Supporting information

S1 Fig Diagram of the tissues examined in animals. Example shown: mouse.
(TIFF)

S2 Fig Histology of the mouth floor from rodent and non-rodent species. Representative mucosal tissue sections (magnification x400) embedded in paraffin and stained with HE are shown.
(TIFF)

S3 Fig Histology of the cheek from rodent and non-rodent species. Representative mucosal tissue sections (magnification x400) embedded in paraffin and stained with HE are shown.
(TIFF)

S4 Fig Collagen fibers density in the mucosa of the mouth floor from rodent and non-rodent species. Representative mucosal tissue sections (magnification x200) embedded in paraffin and stained with Masson trichrome are shown.
(TIFF)

S5 Fig Collagen fibers density in the mucosa of the cheek from rodent and non-rodent species. Representative mucosal tissue sections (magnification x200) embedded in paraffin and stained with Masson trichrome are shown.
(TIFF)
