# Supplementary figures and images for: Comparative analysis of the oral mucosae from rodents and non-rodents: Application to the nonclinical evaluation of sublingual immunotherapy products
Source: PLoS One. 2017 Sep 8;12(9):e0183398. doi: 10.1371/journal.pone.0183398 (PMC5590855; doi:10.1371/journal.pone.0183398)

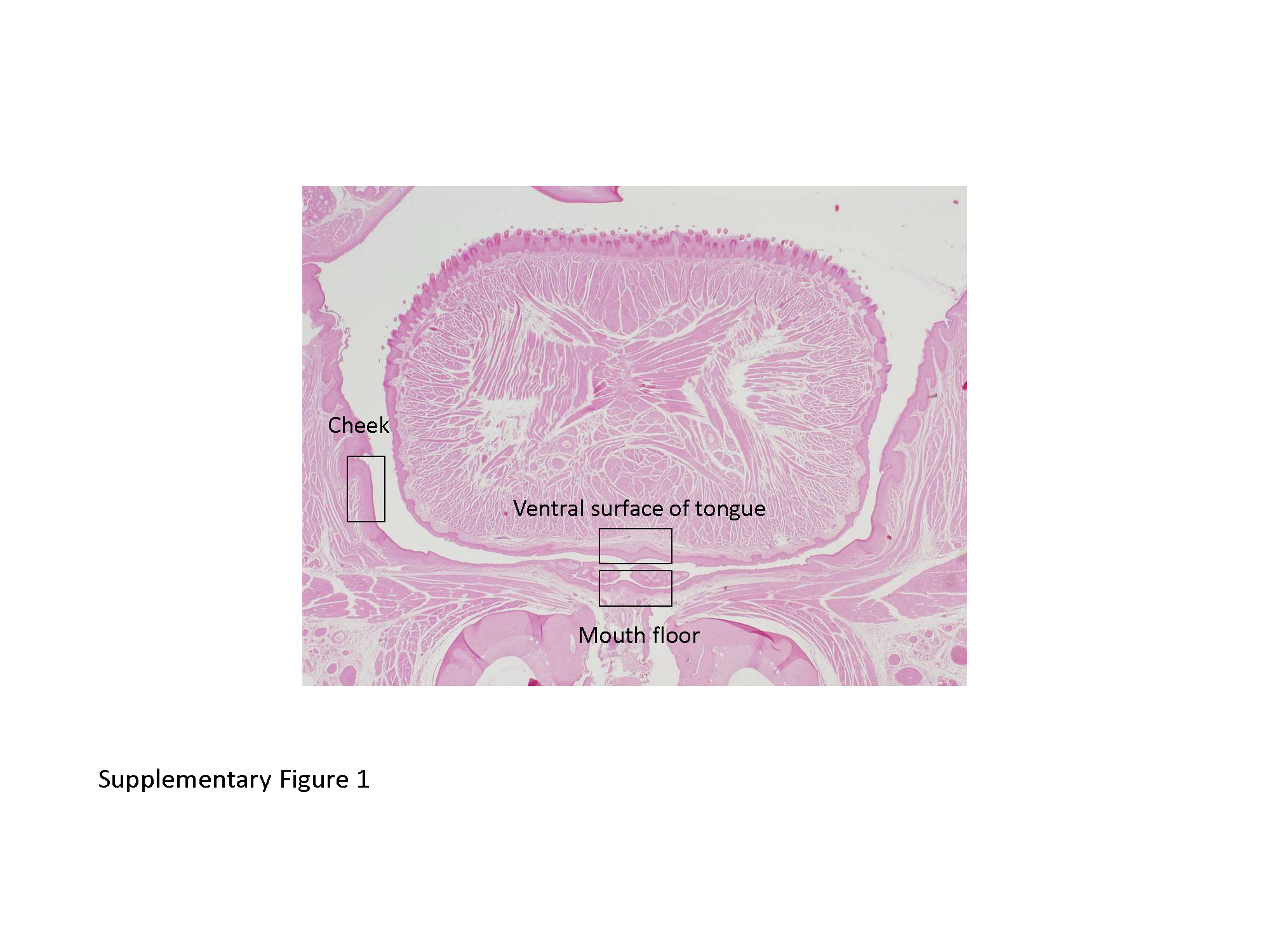

Supplement: S1 Fig — Example shown: mouse. (TIFF) [file pone.0183398.s001.tiff]

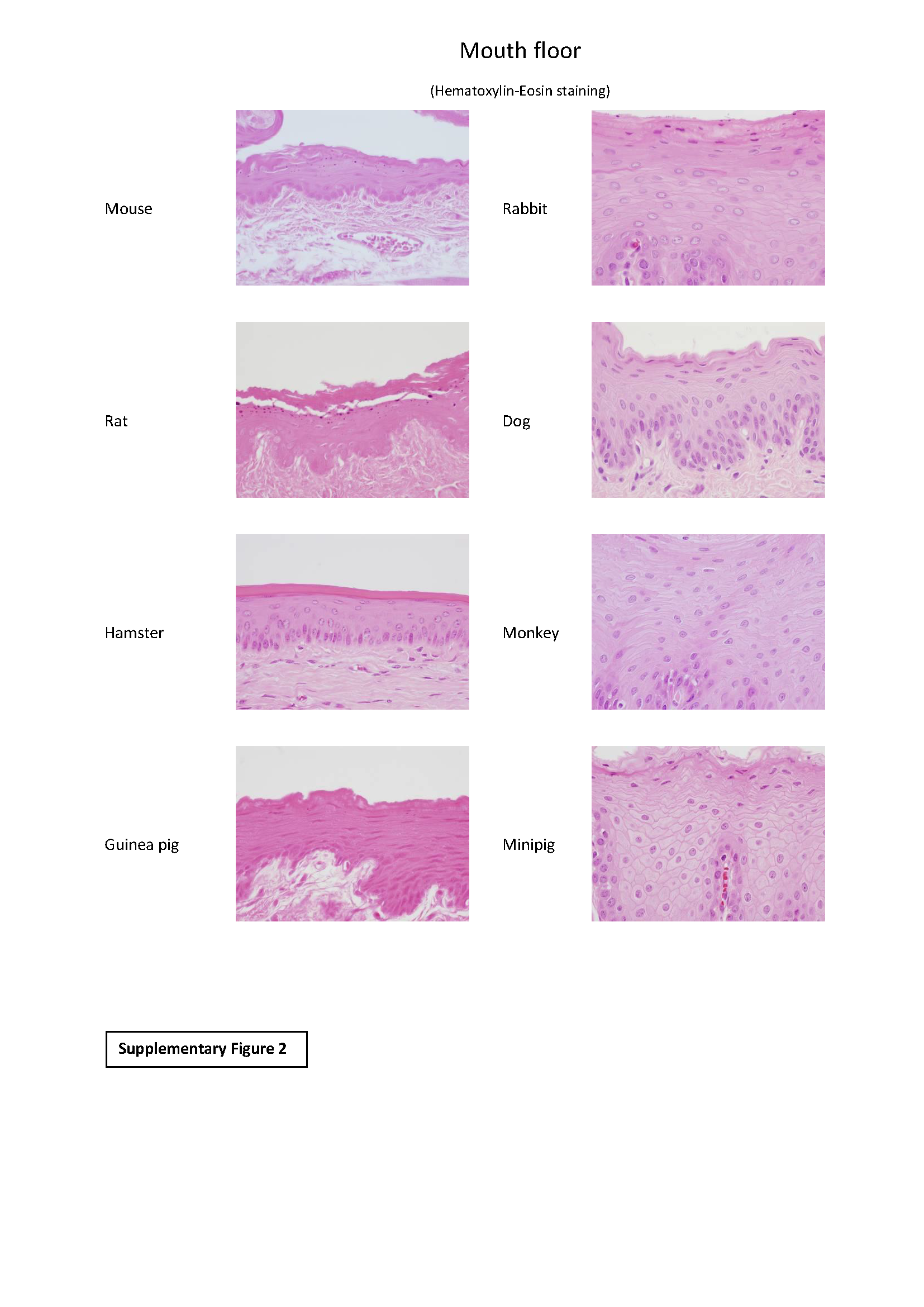

Supplement: S2 Fig — Representative mucosal tissue sections (magnification x400) embedded in paraffin and stained with HE are shown. (TIFF) [file pone.0183398.s002.tiff]

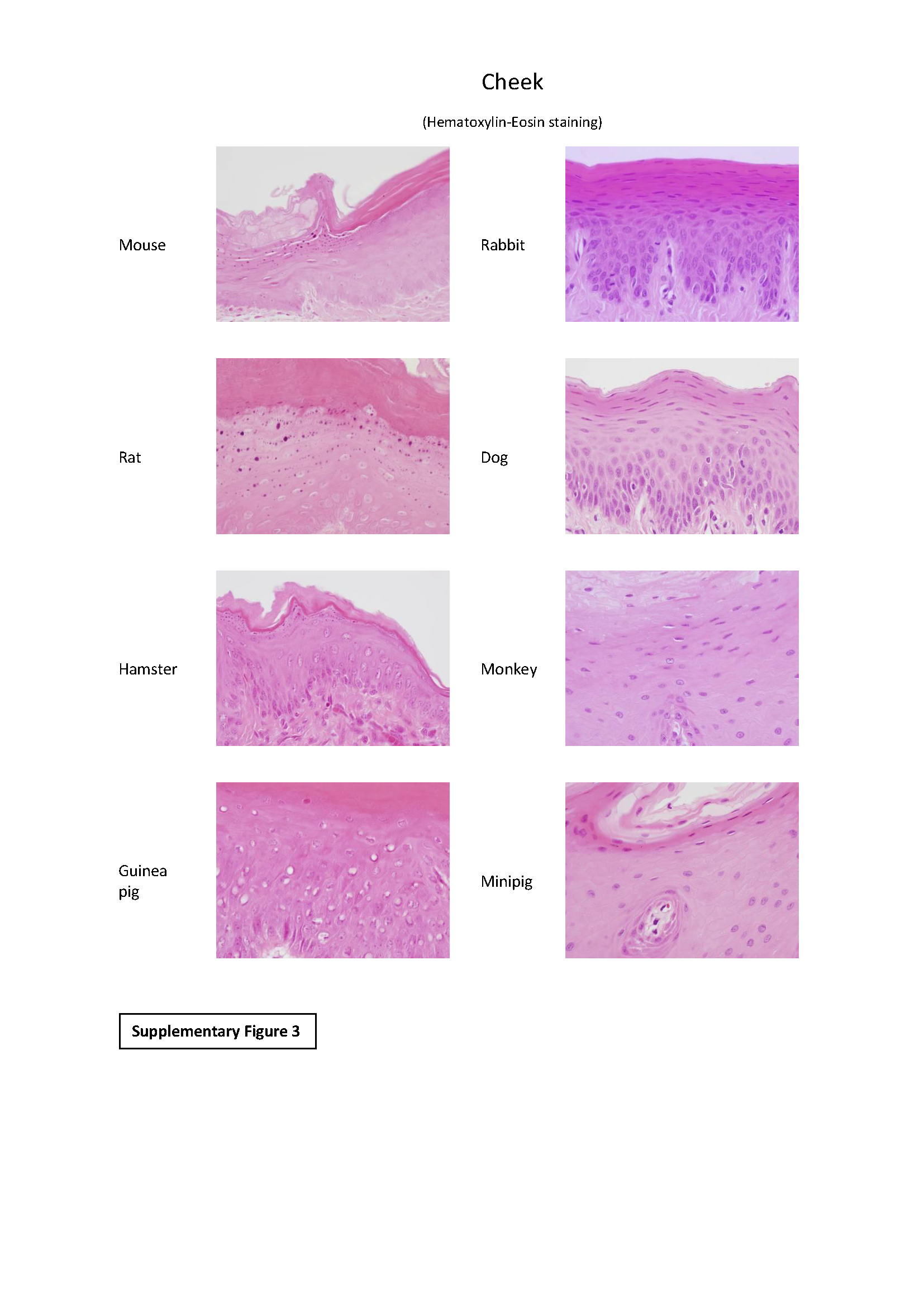

Supplement: S3 Fig — Representative mucosal tissue sections (magnification x400) embedded in paraffin and stained with HE are shown. (TIFF) [file pone.0183398.s003.tiff]

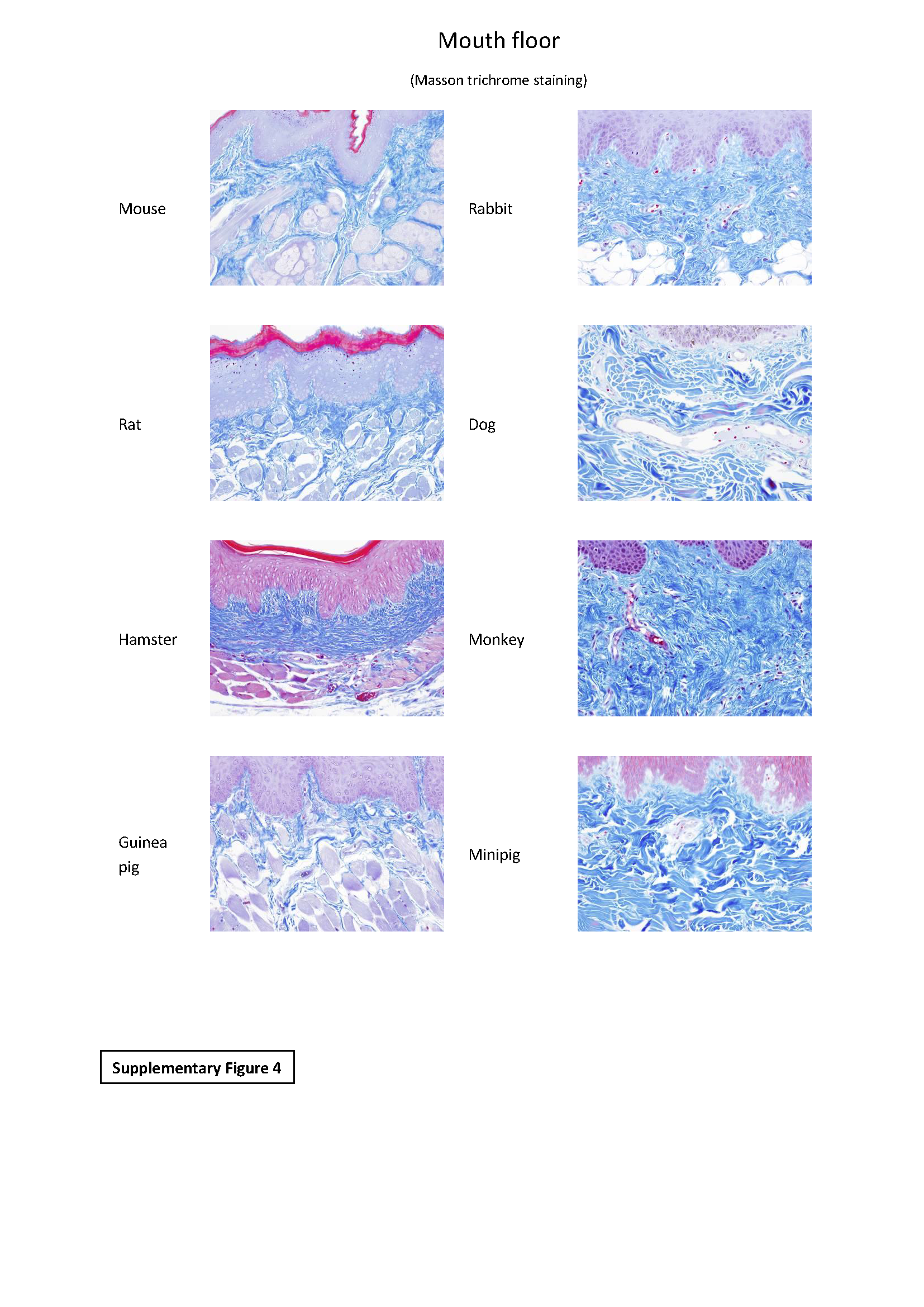

Supplement: S4 Fig — Representative mucosal tissue sections (magnification x200) embedded in paraffin and stained with Masson trichrome are shown. (TIFF) [file pone.0183398.s004.tiff]

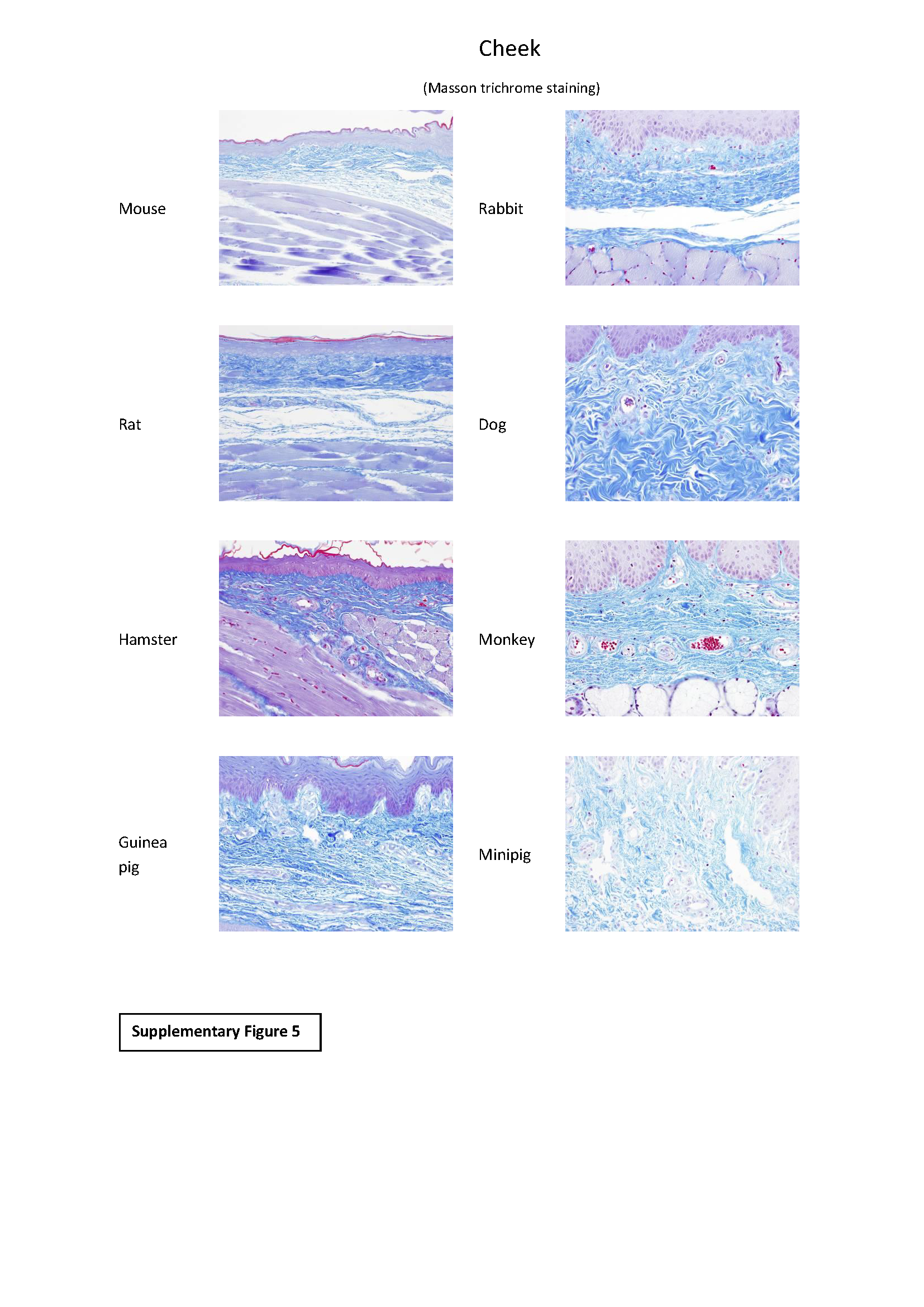

Supplement: S5 Fig — Representative mucosal tissue sections (magnification x200) embedded in paraffin and stained with Masson trichrome are shown. (TIFF) [file pone.0183398.s005.tiff]
